# A new attractant for monitoring western flower thrips, *Frankliniella occidentalis* in protected crops

**DOI:** 10.1186/s40064-015-0864-3

**Published:** 2015-02-24

**Authors:** Zayed S Abdullah, Bethany PJ Greenfield, Katherine J Ficken, James WD Taylor, Martyn Wood, Tariq M Butt

**Affiliations:** College of Science, Swansea University, Singleton Park, Swansea, SA2 8PP UK; Department of Geography, Swansea University, Singleton Park, Swansea, SA2 8PP UK

**Keywords:** Frankliniella occidentalis, Semiochemical, Monitoring, (S)-(−)-verbenone, Thripline-AMS, Sticky trap

## Abstract

Monitoring of pest populations is an essential component of integrated pest management. An early warning system helps growers decide when best to take control measures, or when to alter them, should a control method prove inadequate. Studies have shown that adding chemical attractants to sticky cards can increase trap catch of western flower thrips, *Frankliniella occidentalis*, a global pest of agriculture and horticulture, giving more accurate accounts of population size and dynamics, thus leading to more efficient monitoring. We identify a novel semiochemical to the species, *(S)*-(−)-verbenone, showing that addition of this compound to sticky traps significantly increased *F. occidentalis* catch in two geographically distinct populations, infesting two unrelated crops of global economic importance. We validate through field trials that *(S)*-(−)-verbenone is highly attractive to *F.occidentalis* and can be used with blue sticky traps to enhance trap catch, leading to better estimations of pest population densities. The compound may be used in other control methods against *F.occidentalis* such as lure and kill, mass trapping and push-pull.

**Electronic supplementary material:**

The online version of this article (doi:10.1186/s40064-015-0864-3) contains supplementary material, which is available to authorized users.

## Background

Western flower thrips, *Frankliniella occidentalis* P. (Thysanoptera: Thripidae) is a major agricultural and horticulture pest worldwide (Kirk, 2002; Kirk and Terry, 2003). It causes damage and spoilage to a vast number of economically important plant species through feeding, oviposition and spread of several plant diseases, most notably tospoviruses (Morse and Hoddle, 2006). Their cryptic nature and small size means that they can often remain undetected through quarantine control measures, with their spread having been facilitated by the increase in international plant movement (Kiritani, 2001). A major concern is the rapid development of resistance to conventional chemical pesticides in thrips populations (Jensen, 2000; Bielza *et al*., 2007, Bielza, 2008). The problem is expected to increase since many pesticides have been withdrawn in the EU (Directive 2009/128/EC) highlighting the need to develop alternative control methods for this pest species.

Monitoring of pest populations is an essential component of integrated pest management. An early warning system helps growers decide when best to take control measures, or when to alter them, should a control method prove inadequate. The addition of semiochemicals to monitoring tools increases capture of pests in a wide order of insects (Bhasin *et al.*, 2001; Light *et al.*, 2001; Martel *et al.*, 2005). Using knowledge of pest host specificity and/or preferences, the attractiveness of synthetic host odour blends can be maximised. Addition of optimally attractive chemicals or blends to sticky traps can increase attractiveness and sensitivity, not only providing growers with improved early warning systems, but also facilitating more accurate mapping of population densities and evaluation of the efficacy of control strategies. Such synthetic odours have been developed to enhance monitoring tools for a wide range of insect pests such as the biting midge *Culicoides impunctatus* (Bhasin *et al.*, 2001), codling moth, *Cydia pomonella* (Light *et al.*, 2001) and the colorado potato beetle, *Leptinotarsa decemlineata* (Martel *et al.*, 2005). Furthermore, monitoring allows for the switching of control methods should a particular method cease to provide adequate population suppression, such as in the case of resistance.

Blue and yellow sticky traps are used to monitor thrips population numbers and map their spread within a given area (Shipp 1995; Pearsall, 2002; Broughton & Harrison, 2012). The addition of certain chemical lures to these traps can significantly increase thrips catch, both in fields and glasshouses, sometimes by as much as 100 times (Kirk, 1985; Teulon *et al*., 1993; Murai *et al*., 2000; Imai *et al*., 2001). Increases in trap attraction of *F. occidentalis* as a result of odour lures have been observed on a variety of crop types including but not limited to; pepper (Harbi *et al*., 2013; Teulon *et al*., 2014), bean (Niassy *et al*., 2012; Muvea *et al*., 2014), nectarine (Teulon *et al*., 2014) and strawberry (Sampson and Kirk, 2013). Furthermore, addition of odour lures to sticky traps results in more efficient mass trapping of *F. occidentalis* (Broughton *et al*., 2015), yielding increased economic benefits, particularly with high value crops (Sampson and Kirk, 2013). This paper assesses whether use of the pine pollen volatile *(S)*-(−)-verbenone, previously shown to be attractive to *F. occidentalis* (Abdullah *et al.*, 2014), increases sensitivity and trap catch of *F. occidentalis* on sticky traps in the field, comparing trap catch to two commercially available lures. Thripline-AMS™ developed at Keele University, England (Hamilton and Kirk, 2001), and produced by Syngenta Bioline Ltd (also marketed by Biobest as ThriPher), is a synthetic version of the male *F. occidentalis* pheromone neryl (S)-2-methylbutanoate. Lurem-TR™ is an interspecific kairomone attractant derived from host plants and related compounds (Davidson *et al*., 2007; Teulon *et al*., 2008b), developed by Plant Research International, the Netherlands and Crop and Food Research New Zealand (van Tol *et al*., 2007), and distributed by Koppert Biological Systems, the Netherlands. The semiochemicals differ in their attractiveness to different thrips species. Thripline-AMS is species-specific, attracting male and female *F. occidentalis*, whilst Lurem-TR is interspecific, known to attract *F. occidentalis*, as well as other thrips species (Teulon *et al.,*2008a; Teulon *et al*., 2008b).

## Results

### Release rates of verbenone

**Figure 1:**
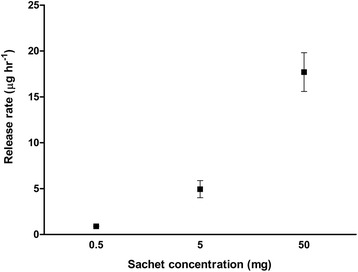
**Release rates as per SPME fibre surface area coverage of release surface of sachets loaded with 0.5 mg, 5 mg and 50 mg** ***(S)*** **-(−)-verbenone in hexane.** Release rates in μg/hour (data presented as mean ± S.E.M).

A total ion chromatogram of *(S)*-(−)-verbenone volatile capture from each sachet loading is included in the electronic supplementary material (Additional file 1). Release rates of verbenone from sachets, as per surface coverage of the SPME fibre of the release surface, can be seen in Figure 1. An average release rate of 0.9 μg/hour was released from the 0.5 mg verbenone loading, 4.9 μg/hour was released from the 5 mg verbenone loading and 17.7 μg/hour was released from the 50 mg verbenone loading. This corresponds to a 1 : 5.4 : 19.7 (0.5 mg : 5 mg : 50 mg) release ratio difference between the different loadings. The absolute release rates in the field would have been much greater, as the method described only samples a relatively small area of the release surface. Furthermore, daily fluctuations in wind speeds and temperature would have resulted in varied release rates at given times (Murlis *et al.*, 1992). Further peak enlargement was observed with a 10 minute exposure to the largest lure concentration, discounting the possibility of fibre saturation. No compounds with a similar retention time were observed in the background air of the laboratory.

### Thrips identification

**Figure 2:**
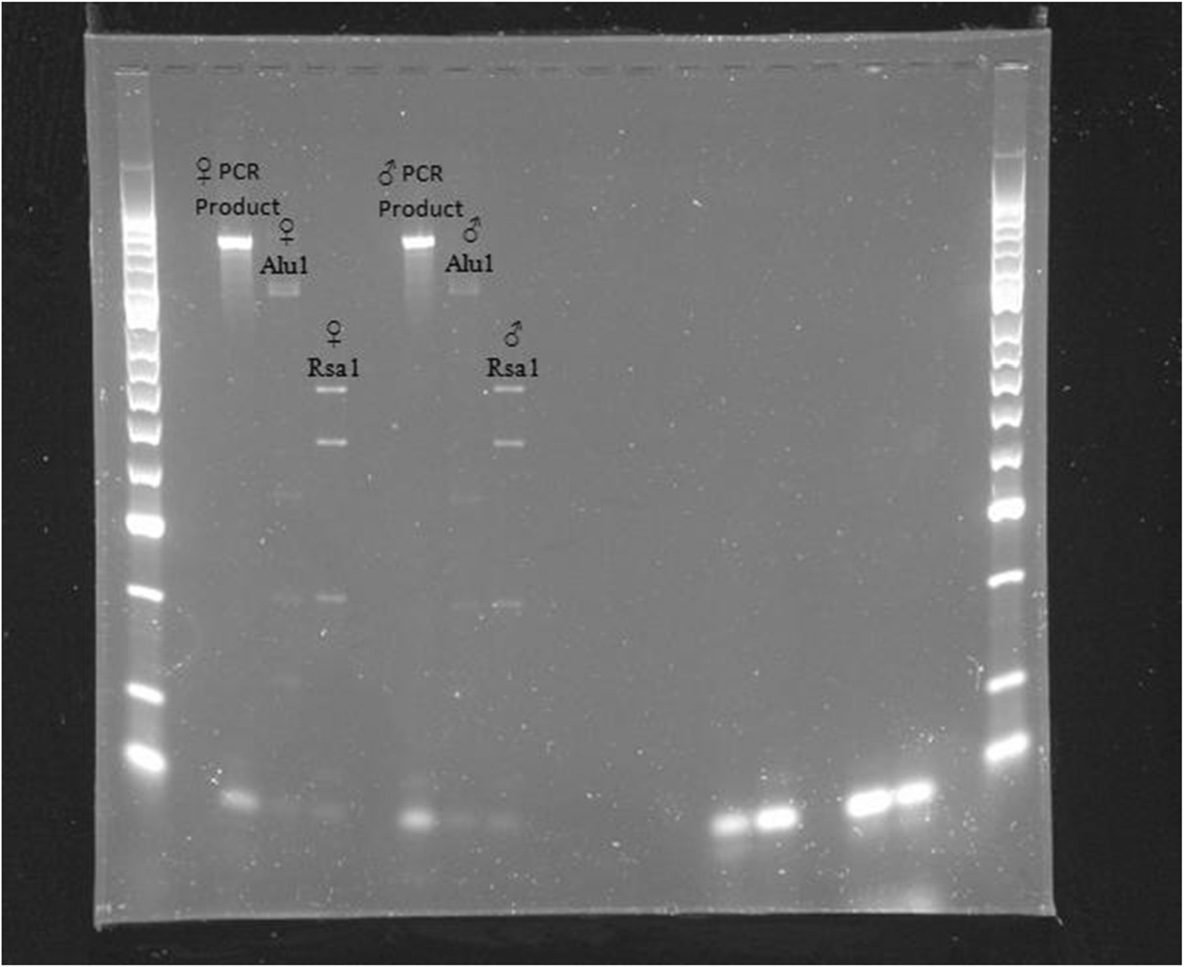
**Gel visualization of band formation from morphologically distinct (smaller and larger) thrips collected in Herefordshire, UK.** Band patterns confirm that both specimens were male and female western flower thrips.

In both experiments, there were primarily two morphologically similar thrips types on the sticky cards and flower tapped samples that were noticeably different in size (Additional file 2), and in the UK experiment, a relatively larger black thrips species was observed, individuals of which were excluded from the counts. Both of the morphologically similar types were found to have red ocelli, antennae comprising of eight antennal segments (with the two end segments being smaller than the others), and a prothorax containing four pairs of the larger ‘strong’ bristles with one pair at each corner, confirming them to be *Frankliniella* spp.. However, the method of identification did not distinguish between species within the genus. Figure 2 illustrates the bands formed from restriction enzyme digestions of the ITS2 region, from the larger and smaller thrips samples collected from Herefordshire, UK. PCR amplification attempts of thrips recovered from the sticky cards were unsuccessful, most likely due to DNA degradation, hence the thrips obtained from the flower tapped samples were used for molecular analysis. The band formation for both types was consistent with *Frankliniella occidentalis*, thus confirming the larger specimens were female and the smaller specimens male, which was supported by the morphological identification. DNA extraction and PCR amplification of samples collected from Turkey were unsuccessful, presumably due to the significant DNA degradation attributable to the longer transportation and storage time before analysis. Therefore, the thrips caught in these trials could not be identified to the species level, and shall be referred to as *Frankliniella* spp.

### Trap catches

**Figure 3:**
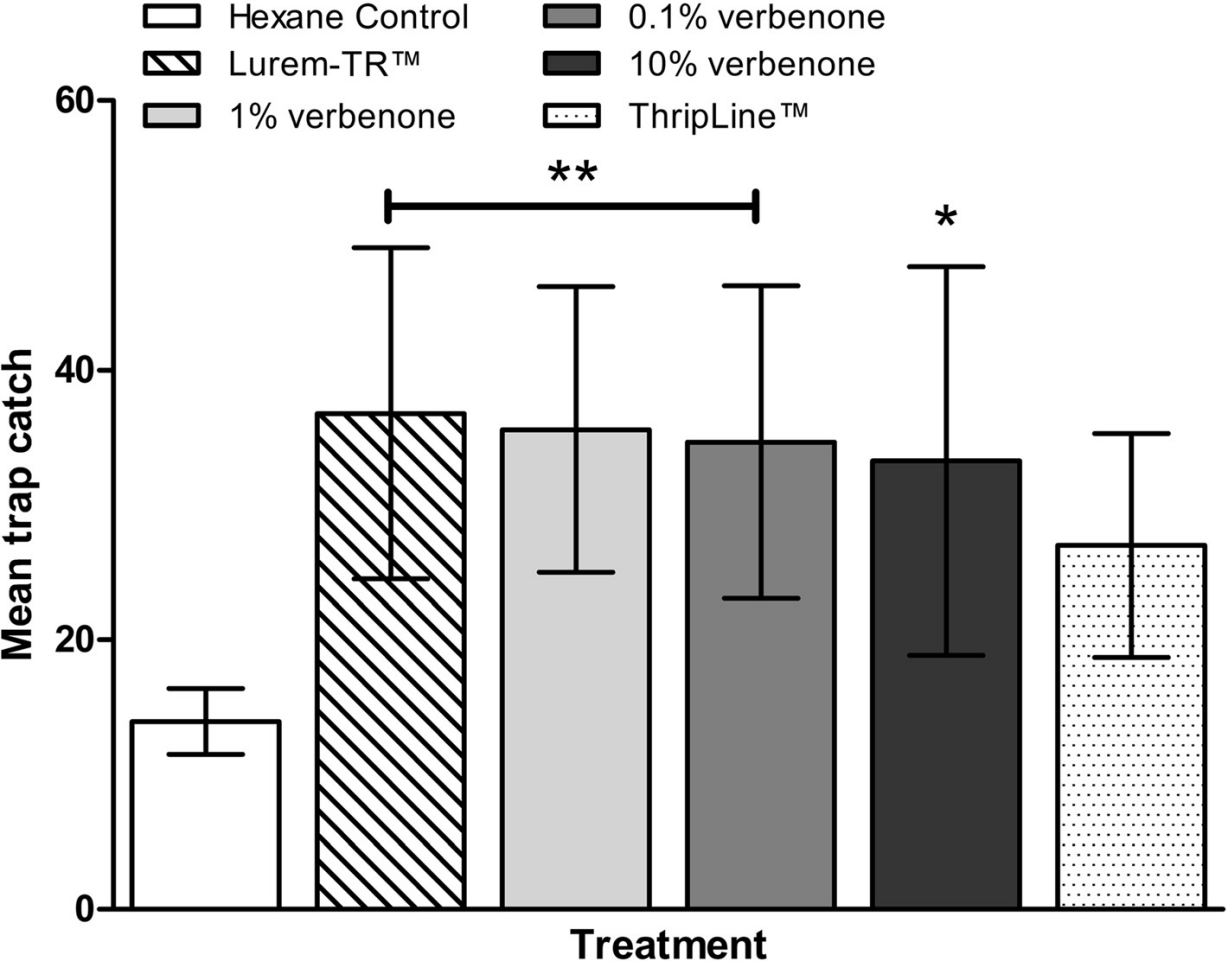
**Data represents untransformed means of 24 hour trap catch of** ***F. occidentalis*** **in Herefordshire, UK.** Square root transformed count data was analysed using a general linear model. Means were separated using Dunnett’s post-hoc, with P = 0.05 considered significantly different when compared to hexane control (data presented as mean ± 95% C.I., * = P < 0.05, ** = P < 0.01, N = 15 per treatment).

Figure 3 illustrates the results from the UK trials. There was no significant difference in trap catch between the Thripline AMS™ treatment and the hexane control (*F*(5,80) = 3.781, p = 0.116). There was a significant increase in trap catch for *(S)*-(−)-verbenone treatments at 0.1% (*F*(5,80) = 3.781, p = 0.008), 1% (*F*(5,80) = 3.781, p = 0.003) and 10% (*F*(5,80) = 3.781, p = 0.029) concentrations. The highest mean trap catch was observed on the sticky traps containing Lurem-TR™ treatment, which was significantly different than the control treatment of hexane alone (*F*(5,80) = 3.781, p = 0.002).

**Figure 4:**
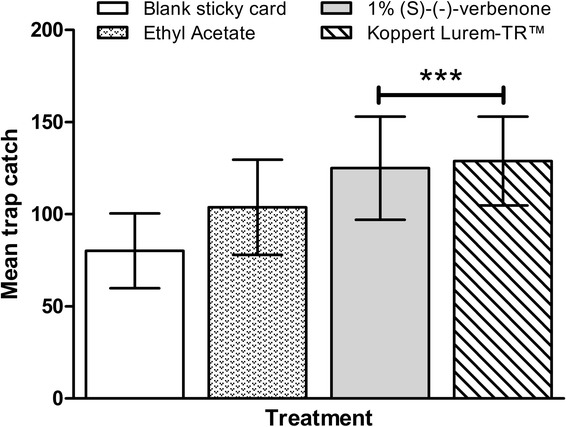
**Data represents untransformed means of 24 hour trap catch of** ***Frankliniella*** **spp. in Antalya, Turkey.** Square root transformed count data was analysed using a general linear model. Means were separated using Dunnett’s post-hoc, with P = 0.05 considered significantly different when compared to blank control (data presented as mean ± 95% C.I., *** = P < 0.001. N = ≥29 per treatment).

Results from the Turkey trials are shown in Figure 4. Three sticky traps were omitted from the experiment, as it was found during the 24 hour count that they had not been adequately setup. These included a blank sticky trap in glasshouse 3, a Lurem-TR™ treated sticky trap in glasshouse 6 and an ethyl acetate treated sticky trap, also in glasshouse 6. In general, higher mean trap catches were observed than in the UK trials. There was no significant difference in trap catch of *Frankliniella* spp. between the blank sticky card treatment and the ethyl acetate treatment (*F*(3,108) = 7.235, p = 0.057). There was a significant increase in trap catch observed for the 1% *(S)*-(−)-verbenone (*F*(3,108) = 7.235, p < 0.001) and Lurem-TR™ (*F*(3,108) = 7.235, p < 0.001) treatments compared to the blank sticky card control.

## Discussion

The results of the UK trials corroborated previous studies that showed application of Lurem-TR™ increases mean sticky trap catches of *F. occidentalis* (Teulon *et al.*, 2007; Broughton and Harrison, 2012; Muvea *et al*., 2014; Teulon *et al.,*^2014^; Broughton *et al*., 2015). The *(S)*-(−)-verbenone treatments significantly increased trap catches at all concentrations, demonstrating the sensitivity of *F. occidentalis* to the compound, as previously demonstrated by electrophysiological and behavioural laboratory based assays (Abdullah *et al.*, 2014). Furthermore, both *(S)*-(−)-verbenone and Lurem-TR™ treatments increased sticky trap catch of two geographically distinct populations of *Frankliniella* spp., infesting two families of economically important crops, indicating that host odours did not reduce the trap catch efficacy of these two semiochemicals for both commodities.

The results in the UK trials were mirrored in the trials in Turkey where the same treatments were applied, even though ethyl acetate was used as a diluent instead of hexane. Ethyl acetate was observed to increase thrips preference to a treatment area within a 4-arm olfactometer, and elicit a significant electrophysiological antennal response (Abdullah *et al.*, 2014), but no significant attraction was observed in the field. Ethyl acetate has been documented to attract a wide order of insects, including but not limited to species of; Diptera (Casana-Giner *et al.*, 1999), Coleoptera (Smilanick *et al.*, 1978), Collembola (Bengtsson *et al.*, 1991) and Hymenoptera (Dicke *et al.*, 1984).

In week one of two of the five experiments carried out by Broughton *et al*., 2015, Lurem-TR™ caught significantly more *F. occidentalis* than Thripline-AMS™ and the control, mirroring the results of the UK trials in this study. In the other two experiments, there were similar trap captures across the Lurem-TR™, Thripline-AMS™ and control treatments, where all treatments caught thrips in relatively less abundance, and in the fifth experiment both Lurem-TR™ and Thripline-AMS™ caught significantly more than the control (Broughton *et al*., 2015). The results of this experiment highlight some variability in the capture potential of the Thripline-AMS™, and the consistency of Lurem-TR™.

In general, higher mean trap catches were observed in the Turkey trials than in the UK trials, and this may have occurred due to a number of reasons. Firstly, the temperatures recorded in the Turkey trials were much higher than those recorded in the UK trials. This may have increased thrips’ populations in the glasshouses in Turkey compared to the polytunnels in UK, resulting in a greater trap catches. Other environmental factors may have also contributed to the increased mean trap catches in turkey, including less wind in the enclosed glasshouses compared to open-ended polytunnels which may have effects thrips’ flight, greater amounts or different types of light reflecting off the sticky traps in the glasshouses compared to the polytunnels as a result of the comprising materials, or the fact that the infested crops in Turkey were better hosts for *F.occidentalis*, therefore resulting in higher population numbers within the plants. Additionally, the efficacy of the traps may have been reduced as a result of interference between treatments in the UK trials due to wind movement patterns through the open-ended polytunnels. As the glasshouses in the Turkey trials were enclosed, wind would movement would have been less likely to result in interferences between treatments.

## Conclusions

We have demonstrated the efficacy of a simple kairomone release sachet, which is cheaper to produce than many commercially available release matrixes, and can be used in combination with sticky traps and semiochemicals to increase trap catches of insects. Verbenone is widely used in perfumery, and can therefore be purchased at lower costs than most thrips pheromone analogues, the production of which requires specialized synthesis. We have shown that such a release sachet impregnated with *(S)*-(−)-verbenone can significantly increase sticky trap catches of *Frankliniella* spp., a globally important pest of agriculture and horticulture, to a level similar to another commercially available product over 24 hours. We validated the efficacy of a novel semiochemical to enhance trap catches of this insect, thus showing its potential as a monitoring tool. The compound may be used in other control methods against *Frankliniella* spp. such as lure and kill, mass trapping and push-pull. We have demonstrated an increase in attractiveness of two geographically disctinct *Frankliniella* spp. when *(S)*-(−)-verbenone is added to sticky traps catch, with pests infesting unrelated commodities of global economic importance. The findings have resulted in the publication of patent WO 2014091185 A1 (Butt and Abdullah, 2014). There is further scope to improve trap catch even more using a blend of other attractive compounds, particularly ones that act on different olfactory receptor neurons. Further evaluations should focus on the range of activity this compound induces in other thrips species, and other insect groups and orders.

## Methods

### Trap design and lures

Blue sticky traps (Suterra, Pontypridd, Wales) measuring 10 cm × 25 cm, containing a black dashed grid on one face, which comprised of 1 cm ^2^ squares were used in this experiment. Counts were made on the gridded area which covered 10 cm × 18 cm of the lower area of the sticky card. The chemical *(S)*-(−)-verbenone (94%, CAS No. 1196-01-6, Sigma Aldrich, UK) was impregnated at different concentrations in ‘Sweet Scent’ sachets (Suterra, Pontypridd, Wales) to determine optimal dose. The sachets were made up of a cellulose pad (6 cm × 1 cm × 0.1 cm) enclosed within a high density polyethylene sachet, which had a 0.3 cm diameter hole in the centre of one surface from which the volatiles emanated. The sachets were made to 0% (500 mg hexane), 0.1% (0.5 mg in 500 mg hexane), 1% (5 mg in 500 mg hexane) and 10% (50 mg in 500 mg hexane) concentrations for the UK trial and 0% (500 mg ethyl acetate) and 1% (5 mg in 500 mg ethyl acetate) for the Turkey trial. The lures were packaged in heat sealed foil sachets (Additional file 3). Commercially available Thripline AMS™ impregnated in rubber septa (Syngenta Bioline, England) and Lurem-TR™ (Koppert Biological Systems, The Netherlands) were used to compare efficacy of verbenone trap catch to presently available commercial lures. The major active component of Thripline AMS™ is the western flower thrips’ aggregation pheromone neryl S-methylbutanoate (Hamilton *et al.*, 2005). The major active ingredient of Lurem-TR™ is the plant kairomone methyl isonicotinate (Teulon *et al.*, 2011). All lures were stored at −20°C or as per manufacturer’s guidelines and were used within a week of receiving. The ‘Sweet Scent’ sachets were stapled to the top of the sticky traps, above the count grid, stapling the outer heat sealed edges of the sachet so as not to affect release rates. The Lurem-TR™ lures were fastened to the plant twist ties used to hang the sticky traps, ensuring that the bottom of the lure positioned roughly in the same region of the trap that the ‘Sweet Scent’ lures were attached. The Thripline AMS™ septa were fitted into the single hole at the top centre of the traps, as per the manufacturer’s guidelines.

### Release rates of lures

#### Volatile collection

Release ratios of *(S)*-(−)-verbenone from lures was calculated using solid phase microextraction (SPME). An exposed 50/30 DVB/Carboxen™/PDMS StableFlex™ SPME fiber (Gray Fiber, Supelco) was laid over the diameter of the sachet hole, resting on the HDPE sachet. The fiber, which was contained in a manual holder, was held in place using a clamp stand, ensuring that the fiber did not come into contact with the saturated cellulose pad within the sachet. The fiber was retracted after 5 minutes of adsorption, after which the volatiles were desorbed and analyzed using gas chromatography coupled mass spectroscopy (GC-MS). A 0.1 μl solution containing 100 ng of *(S)*-(−)-verbenone in hexane was added to the fiber as an in-fiber standard. To ensure that the fiber had not become saturated within 5 minutes of sampling the highest dose lure, an additional 10 minute adsorption was done to ensure further enlargement of the peak, thus ensuring that the fiber had not become saturated. Additionally, to eliminate the possibility of peak enlargement due to *(S)*-(−)-verbenone or compounds with a similar retention time in the laboratory air, a 5 minute adsorption of the room air was carried out at various intervals. 3 replicates were carried out in this fashion for each dosage of lure diluted in hexane and the *(S)*-(−)-verbenone in-fiber standard. An additional replicate was carried out for 1% verbenone diluted in ethyl acetate, to ensure there was no difference in release rate as a result of the diluent.

### Gas chromatography coupled mass spectroscopy

Gas chromatography/mass spectroscopy (GC/MS) analysis was carried out on a HP6890 gas chromatograph coupled to a 5975 inert Mass Selective Detector (Agilent Technologies) operated in electron impact ionization (EI) mode (at 70 eV). SPME fibers were inserted into the GC split/splitless injection port (at 230°C), fitted with a Merlin Microseal (Thames-Resteck, High-Wycombe, UK), operating in splitless mode. Fibers were desorbed for 2 minutes. The GC was fitted with a HP-5MS (J and W Scientific) fused silica capillary column (30 m × 0.25 mm × 0.25 mm film thickness). The oven temperature was held at 40°C for 2 minutes and then increased by 10°C.min^−1^ to 250°C.

### Thrips identification

#### Morphological identification

It was considered impractical to confirm the identity of all of the thrips on the sticky cards due to the high catch numbers; hence only thrips in the bottom left corner of the sticky trap grids were subjected to identification. The thrips were visually identified using a light microscope at ×10 magnification. Thrips were confirmed to be *Frankliniella* spp. only if the Ocelli were observed to be red, the antennae comprised eight antennal segments (with the two end segments being smaller than the others) and the prothorax contained four pairs of the larger ‘strong’ bristles, with one pair at each corner (Moritz *et al.*, 2004).

### Restriction fragment length polymorphism identification

Further identification of thrips was done using restriction fragment length polymorphism (RFLP) analysis of the internal transcribed spacer region 2 (ITS2) of the ribosomal DNA, as per a method described by Moritz *et al.*, 2002.

Comparisons of the base-pair product bands from representative samples were compared to those of confirmed species on a commercially available molecular key (Moritz *et al.*, 2004).

Briefly, DNA was extracted placing ~100 individual thrips from each trial site in a 1.5 ml Eppendorf tube and adding liquid nitrogen. The thrips were ground up to a fine powder using a sterile micro pestle, adding 100 μl of AP1 lysis buffer (DNeasy® Plant Mini DNA Extraction Kit, Qiagen, Netherlands) and heating the tubes at 65°C for 30 minutes. 15 μl of cold 8 M potassium acetate was then added and the tube was incubated on ice for 15 minutes, after which the tubes were centrifuged for 20 minutes at 10,000 × g. The supernatant was transferred to a new 1.5 ml Eppendorf tube and the same volume of isopropanol was added followed by centrifugation at 10,000 × g for 15 minutes. The pellet was washed twice with 70% ethanol, dried, and re-suspended in 16 μl sterile dnase-free water (Qiagen, Netherlands). The ITS2 region was amplified using the primers 28Z 5′AGACTCCTTGGTCCGTGTTTC3′ (Hillis and Dixon, 1991) and P1 5′ATCACTCGGCTCGTGGATCG3′ (Severini *et al*., 1996). Anhydrous primers were purchased from a commercial supplier (Eurofins Scientific, Luxembourg), adding water and storing tubes as per manufacturers guidelines. Each PCR mixture contained 0.5 μl 28Z primer, 0.5 μl P1 primer, 5 μl of extracted DNA solution, 6.5 μl of dnase-free water and 12.5 μl of REDtaq® ReadyMix™ PCR Reaction Mix (Sigma, United Kingdom) to a final volume of 25 μl. The amplification was carried out in a Thermal Cycler (PTC-200 Peltier, MJ Research, USA). The DNA was initially denatured at 94°C for 2 minutes followed by 30 cycles of denaturation at 94°C for 30 seconds, annealing at 55°C for 30 seconds and elongation at 72°C for 2 minutes. The last cycle was followed by a 10 minute incubation period at 72°C to complete any partially synthesized strands. Upon purifying the amplified DNA using a commercial kit (PCR Purification Kit, QIAquick®, Qiagen, Netherlands), RFLP digestions of amplified ITS2 regions were done using the commercially produced restriction enzymes Alu1 (R6281, Promega, USA) and Rsa1 (R6371, Promega, USA), following manufacturers guidelines. Digestion products were visualized on a 1% agarose gel containing 6 μl of gel red nucleic acid stain (10,000× in water, Biotium, USA), comparing products to a 100 bp-2,000 bp ladder (HyperLadder 2, Bioline Reagents Ltd, United Kingdom).

### Field locations and crop types

Two field trials were carried out in separate geographic locations, concomitantly. One field trial was conducted in a set of polytunnels in Herefordshire, UK, at: N 51° 56’ 32.0”, W 2° 41’ 45.2”. A second field trial was conducted in commercial glasshouses in Antalya, Turkey, at: N 37° 01’ 04.05”, E 30°56’ 58.74”.

The UK trial site consisted of a set of open-ended poly-tunnels (7.5 m × 270 m) containing five rows of growing beds with mixed age strawberry plants, *Fragaria × ananassa* var. Elsanta. In Turkey, trials were conducted on pepper plants*, Capsicum annuum* var. Kapya, in enclosed commercial glasshouses (20 m × 80 m). Plants were of similar age within each polytunnel or glasshouse.

### Experimental setup

#### UK poly tunnel trials

Blue sticky traps were hung with plant twist ties (B&Q, UK), 30 m from entrance of the poly tunnel, to ensure even wind distribution across the experimental setup. Sticky traps were hung parallel to one another 50 cm above the outer-most beds, in a randomized block design with a 10 m separation between traps along the right most and left most beds. Five replicate poly-tunnels (blocks) were used with three replicates per treatment. Trials consisted of six treatments, including: hexane sachet (control), three concentrations of *(S)*-(−)-verbenone, Lurem-TR™ and Syngenta Thripline-AMS™ (n = 15 per treatment). Counts were recorded at midday (36°C ±2°C), 24 hr after traps were set.

### Turkey glass house trials

Turkey trials consisted of six replicate glass houses (blocks), with five replicates of each treatment per block. Trials contained four treatments; blank blue sticky traps (control), ethyl acetate only, 1% *(S)*-(−)-verbenone in ethyl acetate and Lurem-TR™ (n = 30 per treatment). Ethyl acetate was used as a diluent in this trial as an alternative to hexane, due to the finding that it was electrophysiologically active and marginally attractive to *F. occidentalis* (Abdullah *et al*., 2014), as well as being less toxic. Sticky traps were hung over the outer-most pepper beds closest to the walls of the glasshouse, with a 7 m separation. Counts were recorded at midday (41°C ±2°C), 24 hr after traps were set.

### Counts

Counts of *F. occidentalis* on cards were made using a tally counter (Scientific Laboratory Supplies Ltd, Nottingham, UK). Only insects caught within the grid area were counted, recording insects caught on both side of the trap. Sticky traps were collected for subsequent morphological and molecular identification of thrips in the laboratory. The presence of thrips species were sampled by collecting >5 individuals in the field, at random, from flowers below each trap in each block, storing the samples in ethanol for subsequent morphological and molecular identification in the laboratory.

### Statistical analyses

Comparison of trap catches with and without lures was analysed using a general linear model (GLM). The model incorporated fixed effects of treatment, including block number as a random factors. Means were compared using a Dunnet’s post-hoc test. Prior to analysis trap catch data was subjected to square root transformation, conforming to GLM assumption of homogeneity of variance, which was confirmed using levene’s test of equality of error variances. All statistical analyses were carried out using SPSS software (IBM Corporation, USA).

## Additional files

Additional file 1:
**Total ion chromatogram of (S)-(−)-verbenone volatile capture from each sachet loading.**
Additional file 2:
**Thrips sample collected from experiment site.**
Additional file 3:
**Sweet Scent’ sachet lure and packaging.**

## Abbreviations

HDPE: High-density polyethylene
GC-MS: Gas chromatography coupled mass spectroscopy
SPME: Solid phase microextraction
RFLP: Restriction fragment length polymorphism
GLM: General linear model
