# Supplementary material for: A new attractant for monitoring western flower thrips, Frankliniella occidentalis in protected crops
Source: Springerplus. 2015 Feb 24;4:89. doi: 10.1186/s40064-015-0864-3 (PMC4348358; doi:10.1186/s40064-015-0864-3)

Additional_file_1 Total ion chromatogram of (S)-(−)-verbenone volatile capture from each sachet loading.

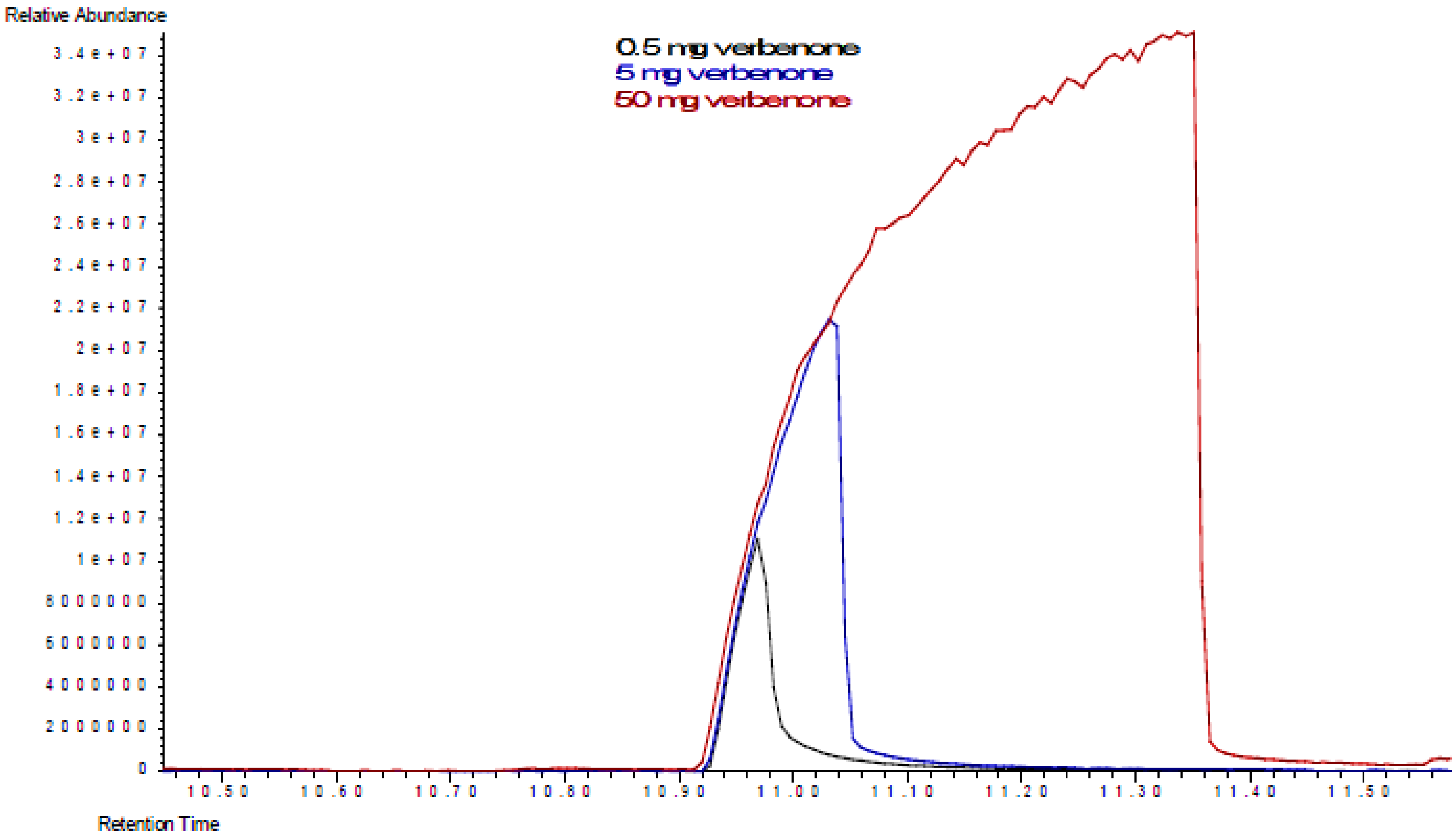

Supplement: Additional file 1: — Total ion chromatogram of (S)-(−)-verbenone volatile capture from each sachet loading. [file 40064_2015_864_MOESM1_ESM.pdf]
