# Supplementary figures and images for: A new attractant for monitoring western flower thrips, Frankliniella occidentalis in protected crops
Source: Springerplus. 2015 Feb 24;4:89. doi: 10.1186/s40064-015-0864-3 (PMC4348358; doi:10.1186/s40064-015-0864-3)

**Additional\_file\_2** Thrips sample collected from experiment site

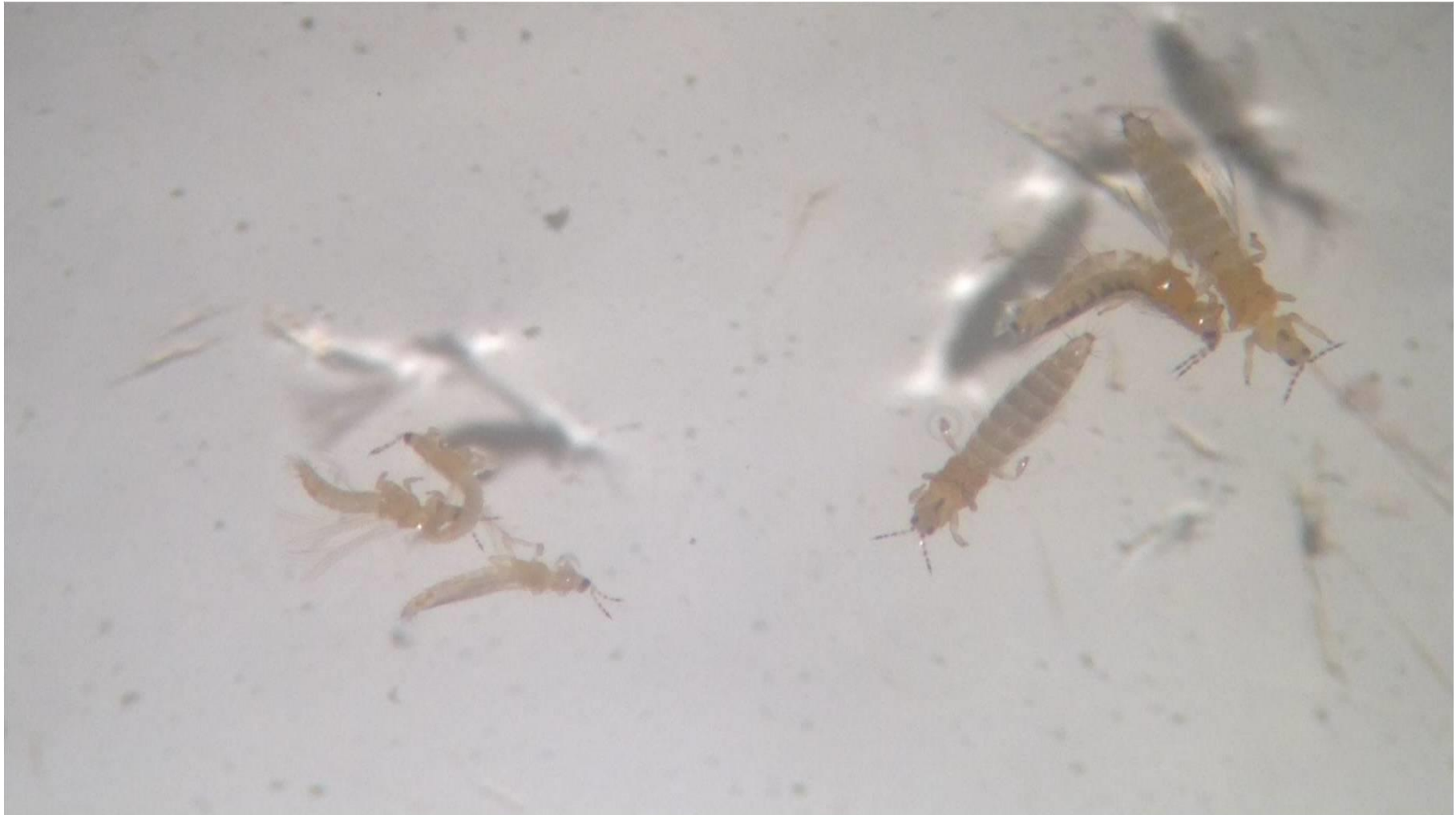

Supplement: Additional file 2: — Thrips sample collected from experiment site. [file 40064_2015_864_MOESM2_ESM.pdf]

**Additional\_file\_3** 'Sweet Scent' sachet lure and packaging

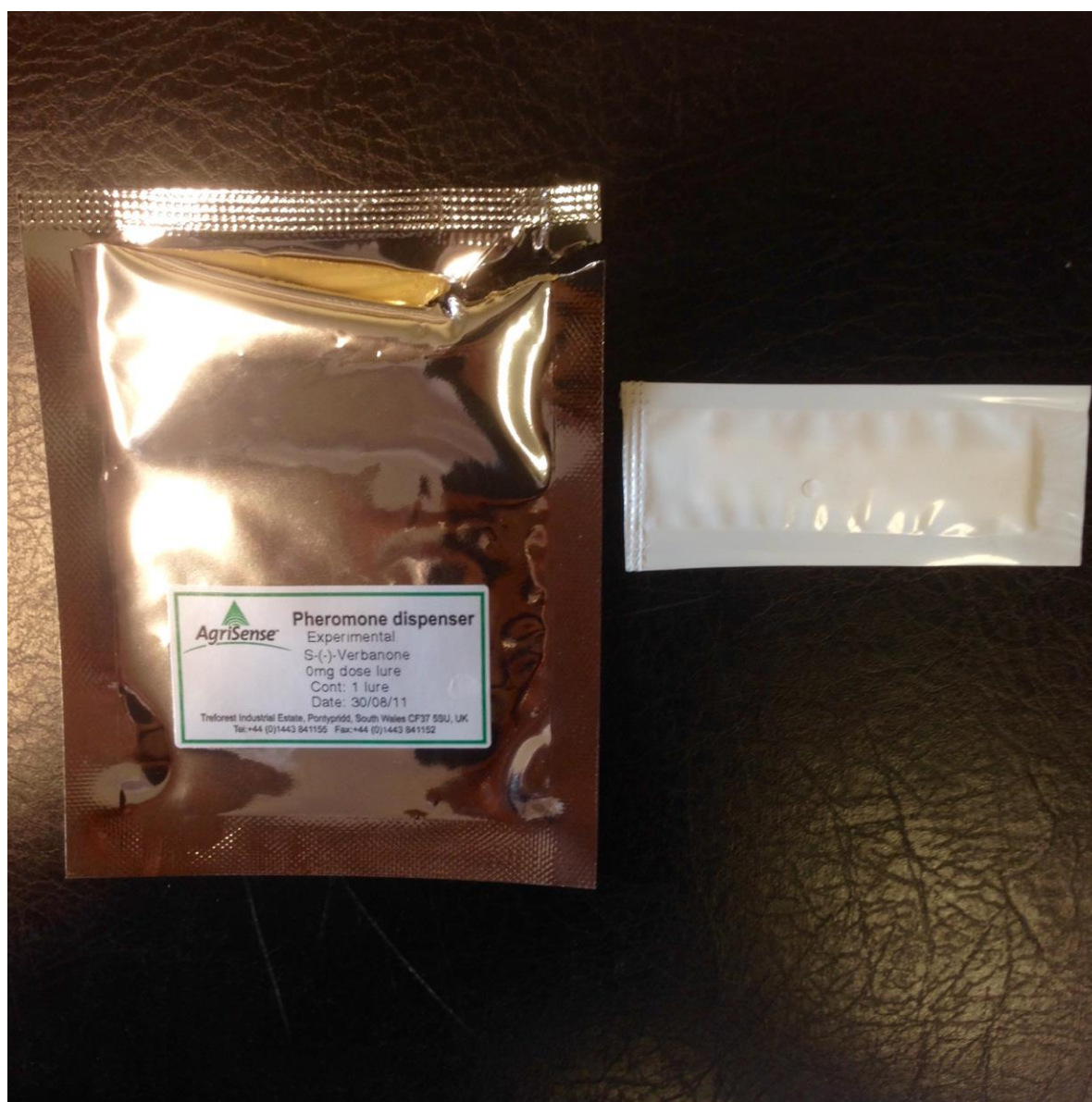

Supplement: Additional file 3: — Sweet Scent’ sachet lure and packaging. [file 40064_2015_864_MOESM3_ESM.pdf]
